# Crotoxin Isolated from *Crotalus durissus terrificus* Venom Modulates the Functional Activity of Dendritic Cells via Formyl Peptide Receptors

**DOI:** 10.1155/2018/7873257

**Published:** 2018-06-03

**Authors:** A. P. Freitas, B. C. Favoretto, P. B. Clissa, S. C. Sampaio, E. L. Faquim-Mauro

**Affiliations:** ^1^Immunopathology Laboratory, Butantan Institute, São Paulo, SP, Brazil; ^2^Department of Immunology, Institute of Biomedical Sciences, University of São Paulo, São Paulo, SP, Brazil; ^3^Physiopathology Laboratory, Butantan Institute, São Paulo, SP, Brazil; ^4^Department of Pharmacology, Institute of Biomedical Sciences, University of São Paulo, São Paulo, SP, Brazil

## Abstract

The *Crotalus durissus terrificus* rattlesnake venom, its main toxin, crotoxin (CTX), and its crotapotin (CA) and phospholipase A_2_ (CB) subunits modulate the immune system. Formyl peptide receptors (FPRs) and lipoxin A_4_ (LXA_4_) are involved in CTX's effect on macrophages and neutrophils. Dendritic cells (DCs) are plasticity cells involved in the induction of adaptive immunity and tolerance maintenance. Therefore, we evaluated the effect of CTX, CA or CB on the maturation of DCs derived from murine bone marrow (BM). According to data, CTX and CB—but not CA—induced an increase of MHC-II, but not costimulatory molecules on DCs. Furthermore, CTX and CB inhibited the expression of costimulatory and MHC-II molecules, secretion of proinflammatory cytokines and NF-*κ*Bp65 and p38/ERK1/2-MAPK signaling pathways by LPS-incubated DCs. Differently, CTX and CB induced IL-10, PGE_2_ and LXA_4_ secretion in LPS-incubated DCs. Lower proliferation and IL-2 secretion were verified in coculture of CD3^+^ cells and DCs incubated with LPS plus CTX or CB compared with LPS-incubated DCs. The effect of CTX and CB on DCs was abolished in cultures incubated with a FPRs antagonist. Hence, CTX and CB exert a modulation on functional activity of DCs; we also checked the involvement the FPR family on cell activities.

## 1. Introduction

Mechanisms of innate and adaptive immunity are triggered by distinct pathogens or pathogenic molecules to restore the individual's physiological condition. In this sense, the innate immunity comprises the first line of defense against pathogens/antigens, and its components are essential for the induction of the proper adaptive immune response [1]. However, some pathogens or products secreted by them are able to modulate/inhibit the immune responses through different mechanisms [2–4].

Dendritic cells (DCs) are considered the link between innate immunity and adaptive immunity participating in the recognition, capture, and presentation of the antigenic peptides to T cell. Therefore, DCs are professional antigen-presenting cells (APCs) that are able to induce naive T cells to differentiate in distinct phenotypes, such as T helper type 1 (Th1), type 2 (Th2), type 17 (Th17), or regulatory T cells (Treg) [5, 6].

The selective interaction of pathogens/antigens (PAMPs) or damaged cells or tissue (DAMPs) by distinct receptors on DCs induces intracellular signaling cascades for activation and maturation or, alternatively, induces the tolerogenic state [7, 8].

As previously described, the recognition of cell wall products from Gram-negative bacteria such as lipopolysaccharide (LPS), via TLR4, promotes DC maturation, which makes these cells able to induce T cell activation [7, 9]. DCs also overexpress the stable complex of peptide/MHC and costimulatory molecules (CD40, CD80, and CD86), which are involved in the prime of naïve T cells [10, 11]. These mature DCs also secrete proinflammatory cytokines such as TNF-*α*, IL-12, and IL-6, which are critical for T cell proliferation and differentiation [12, 13]. The recognition of LPS by TLR4 triggers two major intracellular signaling pathways, one dependent of MyD88 molecule and the other one mediated by Toll/IL-1R domain-containing adaptor-inducing IFN-*β* (TRIF) [14–16]. Both MyD88 and TRIF-dependent pathways downstream involve the sequential activation of distinct intracellular proteins, such as IL-1 receptor-associated kinases (IRAKs) and mitogen-activated protein kinases (MAPKs), culminating with the activation of transcription factors, such as nuclear factor- (NF-) *κ*B and activator protein-1 (AP-1), which act synergistically for the maximal DC maturation and ability to induce T cell priming [14, 15, 17–19].

Otherwise, tolerogenic DCs are able to induce anergy of T cells, clonal deletion or Treg differentiation [20] via production of anti-inflammatory cytokines such as IL-10 [21] and transforming growth factor *β* (TGF-*β*) or expression of inhibitory molecules on the cell surface [22]. In addition to the already-mentioned cytokines that are involved in T cell differentiation, IL-2 is crucial for proliferation, survival, and also differentiation into effector and memory subsets [23]. Considering these observations, the maturation state of DCs is critical to prime the T cells and for induction of adaptive immunity [6].

Crotoxin (CTX) is the main toxin isolated from Brazilian rattlesnake *Crotalus durissus terrificus* (*C*. *d*. *terrificus*) venom. CTX is made up by two noncovalently associated subunits, namely, the crotapotin (CA), that is acidic, nontoxic and enzymatically inactive and a basic subunit (CB), weakly toxic and with phospholipasic A_2_ activity (PLA_2_) [24, 25]. It has been shown that CTX and its subunits have the ability to modulate some parameters of inflammatory reactions, as well as immune response [26].

Regarding immune response, Cardoso and Mota [27] showed an inhibitory effect of CTX on the antibody production in mice immunized with human serum albumin (HSA) or ovalbumin (OVA). It was also demonstrated that CTX is able to modulate leukocyte circulation as well as cytokine and corticosteroid release in mice [28]. A later study has demonstrated that CTX and its CB subunit impair leukocyte circulation in blood and lymph, by increasing the number of B and T lymphocytes in lymph nodes [29].

The work of our group [30] also showed that CTX has the ability to downmodulate the IgG1 and IgG2a antibody production induced by the immunization of mice with human serum albumin (HSA) emulsified in complete Freund's adjuvant (CFA) or adsorbed in Aluminium hydroxide (Alum), even when administered 1 hour before or 3 days after antigen immunization. In addition, it was observed a lower proliferation of T lymphocytes from the mice immunized with HSA/CFA or HSA/Alum and that received the toxin, in response to the stimulation either by HSA or mitogen. As well, it was shown that CTX modulates the macrophage and neutrophil activities and this effect involves lipoxin secretion and the formyl peptide receptors (FPRs) [31–34]. Our group also demonstrated that the increased secretion of lipoxin A_4_ is associated with decreased secretion of cytokines TNF-*α*, IL-1*β*, and IL-6 as well as an increase of CD4^+^FoxP3^+^ population, which are important for the inhibitory effect of CTX on the acute inflammatory intestinal reaction induced by the intrarectal instillation of 2,4,6-trinitrobenzene sulfonic acid (TNBS) in mice [35].

Considering that CTX modulates the immune system and that DCs are the main antigen-presenting cells (APCs) involved in the induction of adaptive immunity or maintenance of the tolerance, in this study, we evaluated the effect of CTX and its subunits on DC maturation and their ability to induce T cell activation.

Our data demonstrated that CTX and CB induce partial maturation of DCs characterized by increased MHC-II expression but not costimulatory molecules. Besides that, the subunit CA does not induce maturation of DCs. In contrast, CTX and CB, but not CA, are able to inhibit the expression of MHC-II and costimulatory molecules as well as the production of proinflammatory cytokines by DCs that were activated with LPS. Furthermore, CTX or CB induced high levels of IL-10, PGE_2_, and LXA_4_ by DCs cultured with LPS. It was also verified that FPRs are involved in this effect of CTX and CB on DC maturation. Lower proliferation of CD3^+^T cells was also verified when cultured with DCs previously incubated with CTX, CB, LPS + CTX, and LPS + CB compared with that obtained in coculture with DCs incubated only with LPS. The CA subunit did not alter the proliferation of lymphocytes incubated with DCs stimulated with LPS.

Taken together, our results demonstrate that CTX and CB subunits exert a modulatory effect on the functional activity of DCs with the involvement of anti-inflammatory mediators and the FPR family in this process, confirming the importance of these mechanisms for immunomodulatory activity of this toxin.

## 2. Materials and Methods

### 2.1. Mice

Male BALB/c and C57BL/6 mice (7-8 weeks of age) were bred in the animal house facilities of the Butantan Institute, São Paulo, Brazil. The animals were kept in temperature (25°C) and humidity-controlled rooms with a 12/12 light-dark cycle and received standard feed and water ad libitum. All procedures were in accordance with the guidelines for animal experimentation and approved by Ethical Committee for Animal Research of the Butantan Institute (CEUAIB, 1005/13; 1311/14).

### 2.2. Antigens and Antibodies

A pool of lyophilized venom from several adult specimens of *C. d. terrificus* snakes was supplied by the Herpetology Laboratory of the Butantan Institute and stored at −20°C at the moment of use.

Lipopolysaccharide from *Escherichia coli* was obtained from Sigma-Aldrich, phosphatase inhibitor cocktail and sodium orthovanadate were obtained from Santa Cruz Biotechnology, and protease inhibitor cocktail was obtained from Roche.

Recombinant murine granulocyte-macrophage colony-stimulating factor (GM-CSF) and recombinant murine IL-4 were obtained from BD Biosciences (CA, USA). Anti-mouse CD3 (clone 17A2), FITC-labeled anti-CD11c (clone N418), PE-labeled anti-class II MHC (I-A^d^), anti-CD80 (clone 1G10), anti-CD86 (clone GL1), anti-CD40 (clone 1C10), and isotype control mAbs were obtained from BD Biosciences (CA, USA).

Antibody anti-mouse for phospho-p38 (Thr180/Tyr182), total p38, phospho-p44/p42 Erk1/2 (Thr202/Tyr204) (clone 20G11), phospho-NF-*κ*B p65 Ser536 (clone 93H1) and total NF-*κ*B p65 (clone C22B4) were the products of Cell Signaling Technology (Beverly, MA). Anti-mouse *β*-actin (clone Poly 6221) was obtained from BioLegend. Anti-mouse Erk 1/2 (p44/p42-clone MK12), goat anti-mouse and anti-rabbit IgG were purchased from Millipore.

Boc-2 (butoxycarbonyl-Phe-Leu-Phe-Leu-Phe) was obtained from Phoenix Pharmaceutical Inc., USA.

### 2.3. Purification of CTX, CA, and CB Subunits

Crotoxin (CTX) was purified from whole venom of the *C. d. terrificus* according to literature [24] with some modifications. In brief, samples of the venom (15 mg) were resuspended in 1.0 mL of Tris-HCl buffer (50 mM, pH 7.0) and subjected to anion exchange chromatography using a Mono-Q HR 5/5 column, previously equilibrated in the same buffer, in an Akta-FPLC system (GE Healthcare, Uppsala, Sweden). The protein content (1 mL/min) was eluted from the column by a linear gradient of NaCl (0-1 M in 50 mM Tris-HCl, pH 7.0).

For the purification of CA and CB, the protocol from other authors [24, 25] was adapted. Briefly, to obtain the CA and CB subunits, a sample of CTX was dialyzed in Tris-HCl (50 mM, pH 7.2) and then was added 0.72 g of urea about 3 mg/2 mL of CTX. The samples were incubated for 18 h at 4°C and after this, subjected to Mono-S HR 5/5 column in a FPLC system. The crotapotin (CA) and PLA_2_ were eluted from the column using a linear gradient of 2.0 M NaCl and 6.0 M urea buffered with Tris-HCl 50 mM, pH 7.2. The fractions were dialyzed to remove salt and urea. The fractions containing CTX and CB were checked for homogeneity and purity by nonreducing SDS-PAGE (12.5%), while the CA was analyzed in 16% polyacrylamide gel in the presence of tricine. The protein content in the samples was determined by the colorimetric method of Bradford.

Samples of CTX, CA, and CB subunits were subjected to polymyxin column according to the protocol provided by the manufacturer (Thermo Scientific Rockford, USA) to remove the endotoxin content (LPS). The endotoxin level in the purified samples was less than 1 (EU) per milligram of protein determined by amoebocyte limulus lysate kit (LAL) (Cambrex/Lonza).

### 2.4. Generation and Stimulation of Bone Marrow-Derived Dendritic Cells (BMDCs)

Immature DCs (iDCs) were generated from the bone marrow of BALB/c mice, as described previously [36] with some modifications. Briefly, femurs were flushed with RPMI-1640 medium and viable cell count was performed by exclusion test using 0.5% trypan blue in a Neubauer chamber. The cells (10^7^/well) were plated out in a 6-well cell culture plate (Costar) in 5 mL of RPMI-1640 medium supplemented (RPMI-S) with 5% fetal bovine serum (FBS), 50 *μ*M 2-ME, 2 mM L-glutamine, 100 mM MEM vitamin solution (Gibco), recombinant GM-CSF, and IL-4 (10 and 5 ng/mL, resp.) and incubated at 37°C in 5% CO_2_. After 4 days, nonadherent cells were washed and a fresh RPMI-S containing GM-CSF and IL-4 was added followed incubation at 37°C in 5% CO_2_. At 7 days, the cells were collected and the purity of CD11c^+^ population was determined using flow cytometry (FACSCanto). The analyses showed 85–90% of CD11c^+^ cells.

The DCs (5 × 10^6^) were stimulated *in vitro* with CTX, CB or CA (250 ng/mL); LPS (250 ng/mL) or LPS + CTX; and LPS + CB and LPS + CA for 18 h at 37°C in 5% CO_2_. In another experiment, DCs were incubated or not with Boc-2 (100 *μ*M) for 15 min at 37°C [37, 38] After this, the cells were washed with RPMI-S and then stimulated with LPS, LPS + CTX, and LPS + CB (250 ng/mL for LPS and toxins) for 18 h at 37°C in 5% CO_2._

### 2.5. Flow Cytometry Analysis

DCs stimulated at different conditions were incubated with anti-Fc*γ*RII/III monoclonal antibody (1 *μ*g/10^6^cells) for 15 min at 4°C followed by centrifugation for 5 min at 500 ×*g*. The cell suspensions (0.5 × 10^6^) were incubated with anti-mouse CD11c, CD80, CD86, CD40, and MHC class II mAb conjugated to FITC or PE in PBS with 1% BSA for 30 min at 4°C. After this, the cells were centrifuged and resuspended in 0.2 mL of PBS with 1% paraformaldehyde. All the cell suspensions were also incubated with PE- or FITC-labeled isotype control mAb. The samples were performed in duplicate (10^4^ events per data acquisition file) and analyzed in the flow cytometer (FACSCanto, BD Biosciences). The data were analyzed with FlowJo software and the results were expressed as the mean ± SD of the mean fluorescence intensity of labeled cells (MFI).

### 2.6. Allogeneic Mixed Lymphocyte Reaction (MLR)

MLR assay was used to evaluate the T lymphocyte proliferation induced by DCs previously incubated at different conditions. The CD3^+^ T cells were purified from periaortic and inguinal lymph nodes of C57BL/6 mice, using MidiMACS columns, according to manufacturer's instructions (Miltenyi Biotech, Germany). For this, lymph node cell suspension was prepared in RPMI1640 medium and incubated with biotinylated anti-CD3^+^ mAb (1 *μ*g/10^6^ cells) for 30 min at 4°C. After this, 5 mL of PBS containing BSA and EDTA was added, followed by centrifugation. The cells were resuspended in 800 *μ*L of PBS containing 0.5% BSA/2 mM EDTA, pH 7.2, with streptavidin microbeads at 20 *μ*L/10^7^ cells followed by incubation for 30 min at 4°C. The cell suspension was washed and diluted in 1 mL of PBS containing 0.5% BSA/2 mM EDTA and subjected to the MidiMACS columns for positive selection, according to the manufacturer's instructions. The purified CD3^+^ T cells were washed and resuspended in complete RPMI.

For MLR assay, DCs (0.6 × 10^5^) previously stimulated with LPS, CTX, CB LPS + CTX, or LPS + CB were cocultured with 3 × 10^5^ CD3^+^ T cells for 72 h at 37°C in a 5% CO_2_ incubator. CD3^+^ T cells or DCs were maintained in medium as negative control. The T cell proliferative response was assessed using Cell Proliferation ELISA Kit version 2 BioTrak system (GE Amersham Healthcare) according to the manufacturer's instructions.

The cultures were performed in quadruplicate and the results were expressed as the mean ± SD.

### 2.7. Cytokine Detection

The cytokine secretion by DCs was measured in the supernatants collected 18 h after stimulation. The IL-12, TNF-*α*, IL-10, IL-2, and IL-6 secretions were determined by ELISA Kits, according to the manufacturer's instructions (e-Bioscience and R&D Systems). The data were expressed as the mean ± SD of the samples in duplicate or triplicate.

### 2.8. Extraction and Quantification of PGE_2_ and LXA_4_

The PGE_2_ and LXA_4_ secretions were assayed in the supernatants from DCs stimulated for 18 h at different conditions. For this, the samples were acidified with 1 N HCl pH 3.4–3.6 and subjected to octadecylsilyl silica column chromatography (C_18_ Sep-Pak columns, Waters® Corporation, USA).

For activating the octadecyl silica column, it was necessary to prewash with 10 mL of water, 2 mL of absolute ethanol and 2 mL of water. After activating the column, the samples were passed and the eicosanoids were eluted from the column with 1 mL of water, 1 mL of ether, and 2 mL of methyl formate. The samples were dried under a stream of nitrogen [37]. The concentration of PGE_2_ and LXA_4_ was determined by competitive-type ELISA (R&D Systems and Neogen, resp.).

### 2.9. Phosphorylation Analysis of p38, ERK1/2, and NF-*κ*B Ser536

Immature DCs were stimulated with LPS in the presence or not of CTX or CB for 5 min. Cells were centrifuged and lysed on ice in RIPA lysis buffer (50 mM Tris-HCl, 150 mM NaCl, 1% NP-40, 0.5% sodium deoxycholate, 0.1% sodium dodecyl sulfate (SDS), pH 8.0) concomitantly with protease/phosphatase inhibitor cocktail and sodium orthovanadate. Protein concentration was determined using a BCA protein assay kit (Pierce Biotechnology) prior to Western blot. Equal amounts of protein lysates (55 *μ*g/well) were mixed with *β*-mercaptoethanol sample buffer. Cells lysates were loaded onto 12.5% SDS-PAGE gels for electrophoresis in a 110 volt constant current, after which resolved proteins were transferred onto nitrocellulose membranes (Bio-Rad) under a current of 0.8 mA/cm^2^ in Trans-Blot Turbo (Bio-Rad). Membranes were washed with Tris-buffered saline (20 mM Tris/150 mM NaCl, pH-7.5) containing 0.1% Tween-20 (TBS-T) for 10 min. After, the membranes were blocked for 1 h at room temperature with 2.5% nonfat dry milk solution in TBS-T and then incubated with primary antibody against phospho-p38, total p38, phospho-ERK p44/42, phospho-NF-*κ*Bp65 Ser536 and total NF-*κ*Bp65 diluted in blocking solution.

In another experiment, samples were blocked for 1 hour in 5% milk/TBS-T and primary for total ERK p44/42 and *β*-actin, both dissolved in the same blocking buffer. Subsequently, the membranes were incubated for 12 hours at 4°C with the antibodies specific for each protein and washed with TBS-T for 30 minutes. After being washed, the membranes were incubated with HRP-goat anti-rabbit or mouse IgG diluted in 2.5% or 5% milk/TBS-T. All samples were incubated for 2 h at room temperature and washed with TBS-T for 30 min.

The immunoreactive bands for phospho-p38, total p38, phospho-ERK1/2 p44/42, phospho-NF-*κ*Bp65 Ser536 and total NF-*κ*Bp65 were detected using an enhanced chemiluminescence detection system (Cyanagen), and for total ERK, p44/42 and *β*-actin were detected using the ECL detection system (Amersham). The bands were visualized on Super RX film (Fujifilm), and densitometric analysis was performed with ImageJ software, version 1.4. Active forms of MAPKs (pERK and p38) and p-NF-*κ*B were normalized with corresponding total nonphosphorylated forms and *β*-actin.

### 2.10. Statistical Analysis

All data were representative of at least two or three independent experiments performed in duplicate or triplicate. The results were expressed as the mean ± standard deviation (SD) of the cytokine and eicosanoid production. The flow cytometry analyses were expressed as the mean florescence intensity (MFI) obtained in DCs incubated at different conditions. Statistical analyses were performed by one-way ANOVA, followed by Tukey's test (GraphPad Prism 5.0, GraphPad Software). *p* values < 0.001 were considered statistically significant.

## 3. Results

### 3.1. CTX and Its CB Subunit Downmodulate the Expression of Costimulatory and MHC-II Molecules on LPS-Stimulated DCs

It has been described that different pathogenic products, such as LPS, are strong inducers of DCs [9]; thus, we investigated the ability of CTX and its subunits to induce DC maturation *in vitro* or modulate the activation of the cells by the LPS. For this, we firstly discarded any cytotoxic effect of CTX, CA, CB, or LPS in DC cultures (data not shown).

**Figure 1:**
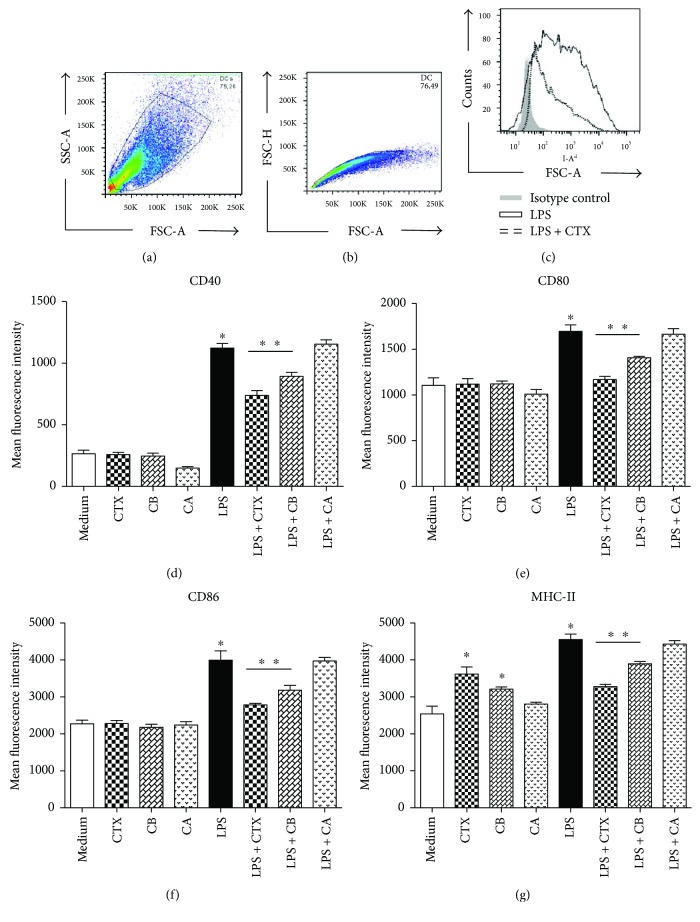
Effect of CTX and its CA and CB subunits on DC maturation *in vitro*. Immature DCs derived from BALB/c mice bone marrow were stimulated with LPS (250 ng/mL), CTX (250 ng/mL), CA (250 ng/mL), CB (250 ng/mL), and CTX/CB/CA + LPS (250 ng/mL + 250 ng/mL) or maintained in culture medium for 18 h. Afterwards, the expression of CD40, CD80, CD86, and MHC-II was assessed by flow cytometry. Firstly, the cells were gated based on size and granularity using forward scatter (FSC) versus side scatter (SSC) to start the analysis (a). Single cells and aggregates were also identified by plotting the FSC width versus FSC area to eliminate debris and clumped cells (b). The representative overlaid flow cytometric histogram shows the expression of MHC-II in DCs stimulated with LPS (black line) or LPS + CTX (dotted line) compared with the cells incubated with isotype control mAb (gray background). The expression of CD40 (d), CD80 (e), CD86 (f), and MHC-II (g) on DCs is showed as the mean fluorescence intensity (MIF) of the samples in quadruplicate ± SD. The results are representative of three independent experiments. Statistical analyses were performed by one-way ANOVA, followed by Tukey's test (GraphPad Prism 5.0, GraphPad Software). ^∗^*p* < 0.001—DCs incubated with LPS compared with DCs maintained in culture medium or incubated with CTX, CB, or CA. ^∗∗^*p* < 0.001—DCs incubated with LPS + CTX or LPS + CB compared with the LPS group. ^∗^*p* < 0.001—DCs incubated with CTX or CB compared with DCs maintained in culture medium.

After this, DCs were preincubated with CTX, CB, and CA (250 ng/mL); LPS (250 ng/mL) or LPS + CTX; and CA or CB (250 ng/mL of each one) for 18 h. At the end of this incubation, we analyzed the expression of MHC-II, CD40, CD80, and CD86 molecules on DCs by flow cytometry, using the gating strategy showed in Figures 1(a)–1(c). The results showed that CTX, CB, and CA did not induce an increase of CD40, CD80, and CD86 expression on DCs when compared with that observed on DCs maintained in RPMI medium (Figures 1(d)–1(f)). However, DCs incubated with CTX and CB showed an enhancement of MHC-II expression in comparison with unstimulated DCs (Figure 1(g)). As expected, the LPS induced higher expression of all molecules evaluated on DCs. However, the CTX and CB subunits, but not CA, were able to inhibit the potent effect of LPS in the expression of MHC-II and costimulatory molecules on DCs *in vitro (*Figures 1(d)–1(g)).

### 3.2. CTX and CB Subunit Interfere with the Secretion of Anti- and Proinflammatory Mediators by DCs

Soluble mediators secreted by different cell populations, such as DCs, exert distinct functions in the innate and adaptive responses. Therefore, the next step was to study the secretion of proinflammatory cytokines by DCs incubated with CTX, CB, and CA in the presence or not of LPS *in vitro*. Furthermore, we also analyzed the secretion of IL-10, PGE_2_, and LXA_2_ in the cell cultures.

**Figure 2:**
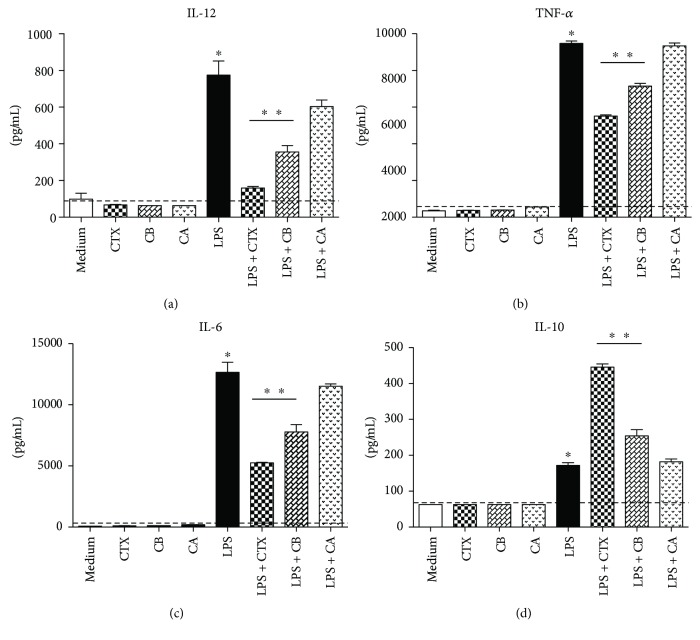
CTX and CB are able to modulate the production of cytokines by DCs incubated with LPS *in vitro.* DCs derived from BALB/c mice bone marrow were stimulated with CTX, CA, or CB (250 ng/mL); LPS (250 ng/mL); and LPS + CTX, CB, and CA (250 ng/mL + 250 ng/mL). After 18 hours, the cell supernatants were collected for detection of IL-12 (a), TNF-*α* (b), IL-6 (c), and IL-10 (d) by ELISA. All samples were performed in quadruplicate and the results were expressed as the mean of the cytokine concentration ± SD. The dashed line represents the detection limit of each cytokine determined from the standard curve. Statistical analyses were performed by one-way ANOVA, followed by Tukey's test (GraphPad Prism 5.0, GraphPad Software). ^∗^*p* < 0.001—groups of cells incubated with LPS compared with DCs maintained in culture medium or DCs incubated with CTX, CB, or CA. ^∗∗^*p* < 0.001—LPS + CTX and LPS + CB groups compared with the LPS group.

Our results show that LPS induced high secretion of IL-12, TNF-*α*, and IL-6 (Figures 2(a)–2(c)) by the DCs compared with that observed in cells maintained in culture medium. In contrast, CTX, CB and CA did not induce the secretion of these cytokines. Furthermore, CTX and CB, when added together with LPS, promoted lower secretion of these proinflammatory cytokines (Figures 2(a)–2(c)) and, in contrast, induced higher production of IL-10 by DCs (Figure 2(d)). We also verified increased expression of TGF-*β* mRNA in DCs incubated with LPS plus CTX or CB (data not shown).

**Figure 3:**
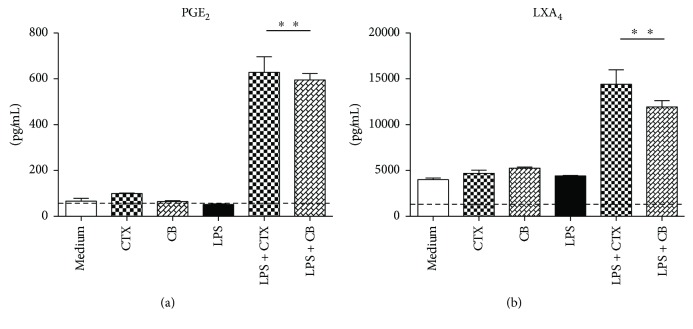
CTX and CB induce an increase of PGE_2_ and LXA_4_ secretion by DCs incubated with LPS *in vitro*. DCs from BALB/c mice were stimulated with CTX, CA, or CB (250 ng/mL); LPS (250 ng/mL); and LPS + CTX, CB, and CA (250 ng/mL + 250 ng/mL). After 18 hours, the cell supernatants were collected for detection of PGE_2_ (a) and LXA_4_ (b) by ELISA. All samples were performed in quadruplicate and the results were expressed as the mean of the eicosanoid concentration ± SD. The dashed line represents the detection limit of each eicosanoid determined from the standard curve. The results are representative of three independent experiments. Statistical analyses were performed by one-way ANOVA, followed by Tukey's test (GraphPad Prism 5.0, GraphPad Software). ^∗∗^*p* < 0.001—LPS + CTX and LPS + CB groups compared with the LPS group.

Considering that CTX induces PGE_2_ and LXA_4_ by macrophages at different experimental conditions [33], we also evaluated these eicosanoids in supernatants of DCs. As show in Figures 3(a) and 3(b), PGE_2_ and LXA_4_ production was increased in DCs incubated with LPS plus CTX or CB compared with the DCs stimulated at all other conditions.

Taken together, these results indicated that CTX and CB, but not CA, are able to modulate DCs maturation induced by LPS.

### 3.3. CTX and CB Inhibit the LPS Signaling Pathways on DCs *In Vitro*

**Figure 4:**
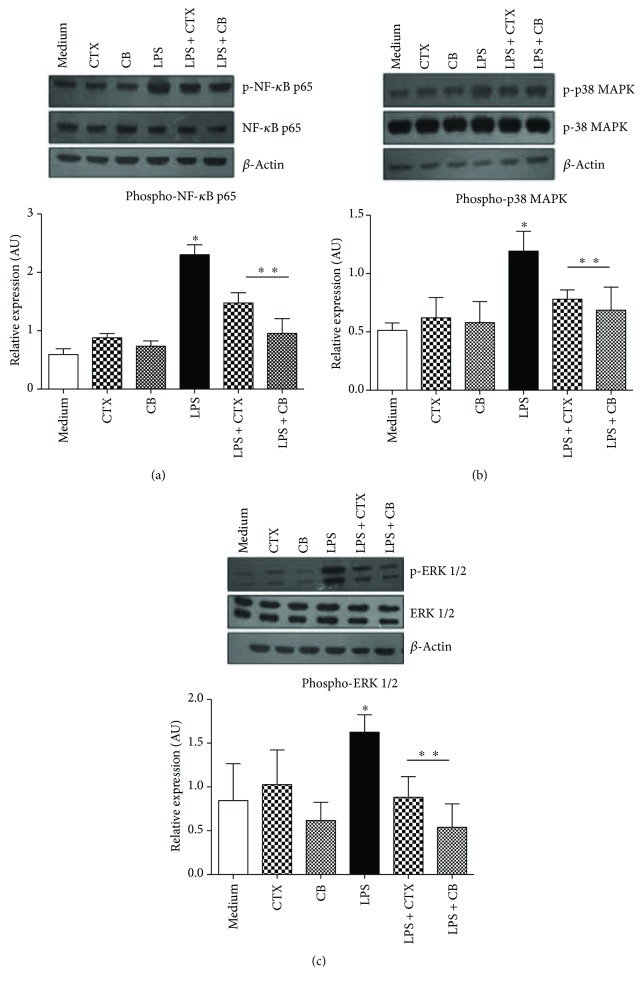
Inhibitory effect of CTX and CB on the activation of NF-*κ*B and MAPK signaling pathways on DCs incubated with LPS *in vitro*. DCs derived from mice bone marrow were stimulated with CTX; CB (250 ng/mL); LPS (250 ng/mL); LPS + CTX; and LPS + CB (250 ng/mL and 250 ng/mL) for 15 min. After this, total cell extracts were analyzed by Western blotting with anti-phospho-NF-*κ*Bp65 and total NF-*κ*Bp65 (a); phospho-p38 and total p38 (b); phospho-ERK1/2 (p44/42) and total ERK1/2 (p44/42) (c); and *β*-actin antibodies. Representative blots were shown and the densitometric analysis of bands was performed by ImageJ software, version 1.44. Values of phospho-ERK1/2, phospho-p38, and NF-*κ*Bp65 were normalized to total ERK1/2, p38 and NF-*κ*Bp65, and *β*-actin and were expressed in arbitrary units ± SD of three independent experiments. ^∗^*p* < 0.001—groups of cells incubated with LPS compared with DCs maintained in culture medium or DCs incubated with CTX or CB. ^∗∗^*p* < 0.001—LPS + CTX and LPS + CB groups compared with the LPS group.

In order to better understand the mechanisms underlying the downregulation of DCs maturation by CTX and CB, we investigated the MAPK (p38 and ERK) and p65 NF-*κ*B activation induced by LPS. Firstly, we observed that both toxins did not induce upregulation of phospho-p38-MAPK, phospho-ERK1/2, or phospho-p65 NF-*κ*B on DCs (Figures 4(a)–4(c)). In accordance with the study of Rescigno et al. [39], LPS induced the phosphorylation of ERK1/2, p38, and p65-NF-*κ*B compared with DCs maintained in culture medium. In contrast, CTX and CB downmodulated the activation of ERK1/2 and p38-MAPKs as well as p65-NF-*κ*B on LPS-incubated DCs (Figures 4(a)–4(c)).

### 3.4. CTX and CB Inhibited the CD3^+^ T Cell Proliferation and IL-2 Production in MLR

**Figure 5:**
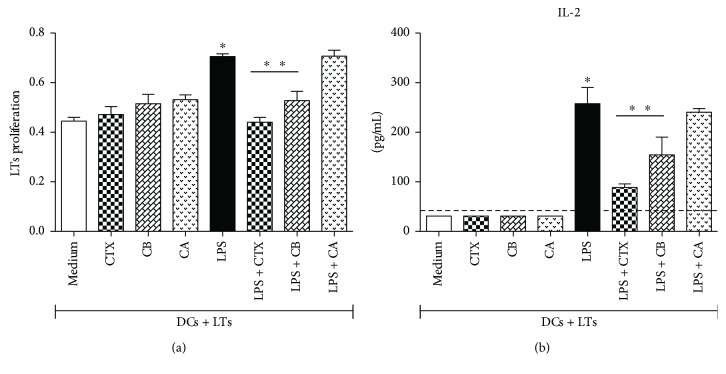
CTX and CB inhibit the proliferative response and IL-2 production of CD3^+^ T cells cocultured with DCs preincubated with LPS. DCs differentiated *in vitro* from bone marrow of BALB/c mice were incubated for CTX, CB, and CA (250 ng/mL); LPS (250 ng/mL); and LPS + CTX, LPS + CB, and LPS + CA (250 ng/mL and 250 ng/mL) for 18 h. The cells (0.6 × 10^5^) were cocultured with CD3^+^ lymphocytes (3 × 10^5^) purified from C57BL/6 mice. As controls, DCs and CD3^+^ cells were maintained in culture medium (date not shown). (a) The proliferative response was analyzed at 72 h of culture as described in Materials and Methods. (b) The coculture supernatants were collected for IL-2 detection by ELISA. The results were expressed as the mean of the optical density obtained from the samples in quadruplicate ± SD for the lymphocyte proliferation assay. The data are representative of two independent experiments. IL-2 secretion was expressed as the mean of the samples in triplicate ± SD. The dashed line represents the detection limit of the assay. Statistical analyses were performed by one-way ANOVA, followed by Tukey's test (GraphPad Prism 5.0, GraphPad Software). ^∗^*p* < 0.001—CD3^+^ T cells plus DCs incubated with LPS compared with CD3^+^ T cells plus DCs previously incubated with medium, CTX, CB, or CA; ^∗∗^*p* < 0.001—CD3^+^ T cells plus DCs incubated with LPS compared with CD3^+^ T cells plus DCs stimulated with LPS + CTX or LPS + CB.

Previous results indicated that CTX and CB were able to inhibit the maturation of DCs stimulated with LPS. Therefore, we investigated whether this modulation should be relevant for the ability of the DCs to induce T cell proliferation. Hence, we analyzed the TCD3^+^ proliferation in MLR and the results showed high T cell proliferation and IL-2 secretion in cocultures with DCs previously incubated with LPS or LPS plus CA. In contrast, lower proliferative response as well as IL-2 secretion were verified in cocultures of CD3^+^ cells and DCs previously incubated with LPS plus CTX or LPS plus CB compared with that observed in cocultures of CD3^+^ cells with DCs incubated with LPS. Furthermore, we also verified low proliferation and IL-2 secretion in cocultures of CD3^+^ with DCs previously incubated with medium, CTX, CA, or CB, as showed in Figures 5(a) and 5(b).

### 3.5. Involvement of FPRs in the Modulatory Activity of CTX and CB Subunit in LPS-Induced DC Maturation

**Figure 6:**
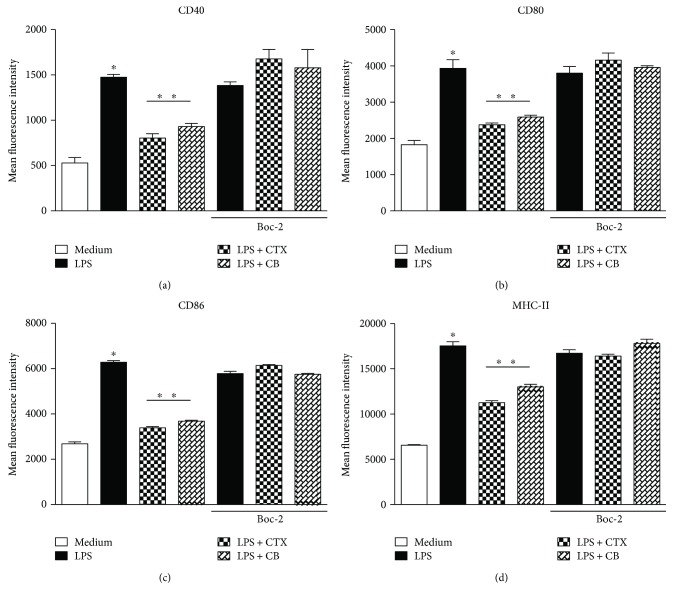
FPRs are involved in the modulatory effect of CTX and CB on the DC maturation induced by LPS. DCs differentiated *in vitro* from bone marrow of mice were incubated or not with Boc-2 (100 *μ*M) for 15 minutes. After this, the cells were centrifuged and added with the culture medium containing LPS (250 ng/mL), LPS + CTX, or LPS + CB (250 ng + 250 ng/mL) for 18 h. After this, the cells (10^6^) were labeled with anti-CD40 antibody (a), anti-CD80 (b), anti-CD86 (c), and anti-MHC II (d) and analyzed by flow cytometer. DC samples were also incubated with anti-CD11c-PE antibody or PE isotype control. The results were expressed as the mean fluorescence intensity (MIF) of DCs in quadruplicate ± SD. The data are representative of two independent experiments. Statistical analyses were performed by one-way ANOVA, followed by Tukey's test (GraphPad Prism 5.0, GraphPad Software). ^∗^*p* < 0.001—groups of DCs incubated with LPS compared with cells maintained in culture; ^∗∗^*p* < 0.001—LPS + CTX and LPS + CB groups compared with the LPS group.

**Figure 7:**
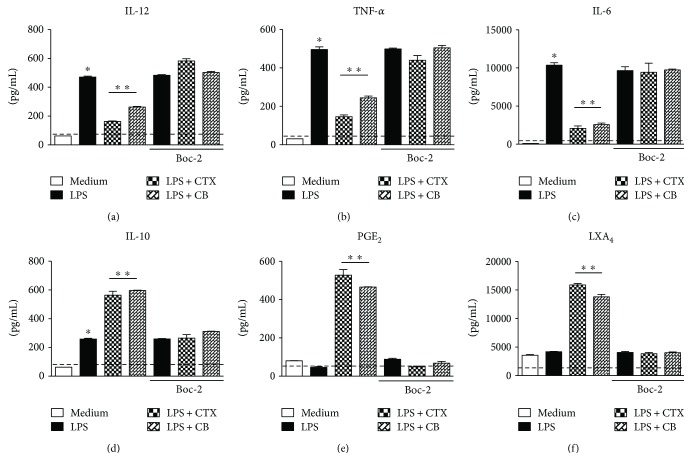
Role of FPRs in the modulatory effect of CTX and CB on cytokines and eicosanoids secretion by DCs incubated with LPS. DCs differentiated *in vitro* were incubated or not with Boc-2 (100 *μ*M) for 15 minutes. After this, the cells were centrifuged and added with the culture medium containing LPS (250 ng/mL), LPS + CTX, or LPS + CB (250 ng + 250 ng/mL) for 18 h. After this, the cell supernatants were collected for measuring of IL-12 (a), TNF-*α* (b), IL-6 (c), IL-10 (d), PGE_2_ (e), and LXA_4_ (f) secretion by ELISA. The results were expressed as the mean of the cytokine concentration of each sample in quadruplicate ± SD. The dashed line represents the detection limit of the each assay. ^∗^*p* < 0.001—groups of DCs incubated with LPS compared with cells maintained in medium culture; ^∗∗^*p* < 0.001—LPS + CTX and LPS + CB groups compared with the LPS group. The data are representative of two independent experiments. Statistical analyses were performed by one-way ANOVA, followed by Tukey's test (GraphPad Prism 5.0, GraphPad Software).

Considering our results and that the FPR family is involved in the modulatory effect of CTX on some macrophage activities [37], our next aim was to analyze the role of these receptors on the downmodulatory effect of CTX and CB on LPS-stimulated DCs. For this, DCs were incubated or not with Boc-2, a selective FPR antagonist, followed with incubation with LPS, LPS plus CTX, or LPS plus CB. In these cells, we evaluated the expression of costimulatory and MHC-II molecules and the secretion of cytokines and eicosanoids. The results showed again that LPS induced an increase in the expression of MHC-II and costimulatory molecules (Figures 6(a)–6(d)) as well as the proinflammatory cytokine secretion (Figures 7(a)–7(c)) by DCs incubated or not with Boc-2. The downmodulatory effect of CTX and CB on DCsmaturation induced by LPS was also verified; however, the addition of Boc-2 in DCs reverted these effects (Figures 6(a)–6(d) and 7(a)–7(c)). Furthermore, we also observed higher secretion of IL-10, LXA_4_, and PGE_2_ in DCs incubated with LPS plus CTX or LPS plus CB when compared with DCs incubated with LPS or maintained with culture medium in the absence of Boc-2. However, the IL-10, PGE_2_ and LXA_4_ productions were abolished when the Boc-2 was added to the DCs incubated with the LPS plus CTX or CB (Figures 7(d)–7(f)). Therefore, the data indicate the participation of FPRs in the modulatory effect of CTX and CB on DCs.

## 4. Discussion

Dendritic cells (DCs) are considered as potent and highly versatile antigen-presenting cells promoting the protective adaptive immunity as well as contributing to tolerance. However, the mechanisms involved in this plasticity of DC activities have been the focus of investigations [40, 41]. Besides the classical biological activities of the crude *C. d. terrificus* venom in the envenomation, a modulatory property of the crude venom, the main toxin-CTX, or its subunits (CA and CB) on the immune system has been described [26, 29]. However the effect of these molecules on the DCs had not been previously investigated. Our results demonstrate that CTX and CB induce partial maturation of DCs and also exert a potent inhibitory effect on LPS-induced DC maturation. In addition, the FPRs are involved in the modulatory effects of CTX and CB on the functional activity of DCs.

Immature DCs are found in peripheral tissues, where they are exposed to continuous environmental signals. Since these cells recognize antigens or inflammatory signals, such as activation of TLRs, they differentiate into mature cells, which comprise the high expression of MHC/peptide complexes and costimulatory molecules, such as CD80 and CD86, and chemokine receptor CCR7. Thus, these cells migrate to lymphoid tissue and then present the antigen to T lymphocytes [8, 11, 40, 41].

In this sense, it has been shown that microbial products are able to induce DC activation and maturation or, when added together with a low immunogenic antigen, able to present the adjuvant property to potentiate the antigen-specific DC maturation and secretion of proinflammatory cytokines [2, 42]. On the other hand, some antigens derived from pathogens can inhibit the activation of DCs such as products of helminthes and bacteria [2–4].

Therefore, we firstly evaluated the effect of CTX and its subunits on DC maturation. For this, we used a previously described *in vitro* model of DC differentiation from the bone marrow of mice using GM-CSF and IL-4 [36]. Our results showed that CTX and CB, but not CA, induced an increase of MHC-II but not the costimulatory molecules on DCs when compared with that observed in DCs maintained in culture medium. In addition, CTX, CB, or CA did not stimulate the secretion of IL-6, TNF-*α*, and IL-12 by DCs.

As previously showed [9], we also verified that LPS promoted a potent stimulatory effect on the expression of MHC-II, CD40, CD80, and CD86 on DCs, as well as the secretion of proinflammatory cytokines. In contrast, the addition of CTX and CB during DC incubation with LPS markedly impaired the upregulation of MHC-II and costimulatory molecule expression and also the cytokine secretion by these cells. The CA subunit, in turn, was not able to prevent the maturation of DCs induced by LPS.

As mentioned, the DC maturation triggered by LPS involves the MyD88- and TRIF-dependent signaling pathways downstream [15] with consequent activation of IRAKs, and MAPKs, such as JNK, p38, and ERK1/2 as well as NF-*κ*B are involved in the upregulation of inflammatory cytokines, expression of type I IFNs, MHC class I/II, and costimulatory molecules [14, 16, 43]. In our results, the analysis of the effect of CTX and CB on the LPS signaling pathways on DCs allowed us to observe that both molecules were able to inhibit the phosphorylation of NF-*κ*Bp65 and MAPKs, such as ERK1/2 and p38 in DCs incubated with LPS. Therefore, these data are in agreement with the inhibitory effect of CTX and CB on the expression of costimulatory molecules and MHC-II, as well as the secretion of proinflammatory cytokines by DCs incubated with LPS.

Moreover, our results are also in agreement with those reported by Sampaio et al., which described that the modulatory activity of CTX on the macrophages spreading and phagocytic activities is mediated by the CB subunit. Furthermore, high production of PGE_2_ and LXA_4_ by macrophages incubated with crude *C.d. terrificus* venom, CTX or CB subunit was showed [32, 33, 44]. The PGE_2_ production has been correlated with the induction of human Treg [45].

Zhang et al. [46] showed that LXA_4_ is able to inhibit the differentiation of RAW264.7 murine macrophages to dendritic-like cells by stimulation with LPS. The authors also demonstrated that LXA_4_ abrogated the upregulation of MHC-II and CD86 expression and also the allogeneic stimulatory activity of DCs derived from LPS-stimulated RAW264.7 cells. In another work [47], the induction and regulation of IL-12 secretion by DCs using the *in vivo* model of injection of an extract of tachyzoites from *Toxoplasma gondii* (STAg) were studied. The results showed that the high secretion of IL-12 by splenic DCs in mice injected with STAg, as observed in the initial of parasite infection, was abolished in a second STAg injection. The data also demonstrated that this DC's unresponsiveness to microbial stimulation was mediated by the increased secretion of LXA_4_ and downregulation of CCR5 by a mechanism dependent of G-protein coupled receptors, such as FPRs.

Another work also shown that IL-10 and TGF-*β* are produced by anti-inflammatory DCs [48]. Therefore, we analyzed these cytokines in our DC cultures and the results showed an increase of IL-10 secretion and expression of TGF-*β* (data not shown) by DCs incubated with LPS plus CTX or CB. Again, the addition of CA did not interfere with these cytokines.

The differentiation stages of DCs are directly correlated with their role into T cell immunity or tolerance. In this context, several observations support the hypothesis that the immature or partially mature DCs are closely related to the induction of tolerance and Treg differentiation, whereas the fully mature DCs induce T cell immunity. The phenotypic state of development or activation of DCs is distinguished by the expression of MHC and costimulatory molecules and secretion of cytokines. The fully mature DCs are MHC-II^high^costimulatory^high^ and produce large amounts of proinflammatory cytokines, mainly IL-12. On the other hand, the IL-10 production by partially mature DCs has been described [49]. Therefore, our results suggest that CTX and its subunits are not inducers of DC maturation as observed for a wide range of microbial products. In addition, CTX and CB impair the full maturation of DCs induced by LPS, possibly maintaining these cells in a partially mature stage.

Since the appropriate T cell activation by DCs is dependent of the high expression of costimulatory molecules and antigen/MHC complexes [50, 51], we then evaluated the ability of the DCs incubated at different conditions to induce allogeneic T cell proliferation. Our data showed that the DCs incubated with CTX, CA or CB did not induce a significant T cell proliferation compared with DCs maintained in culture medium. We also observed an increased proliferation of T cells cocultured with LPS. In contrast, the addition of CTX or CB, but not CA, in DCs incubated with LPS cocultured with T cells resulted in inhibition of the proliferation compared with that induced by LPS-matured DCs. We also analyzed the secretion of IL-2, which is directly correlated with T cell proliferation and observed a lower secretion of this cytokine in cocultures of CD3^+^ T cells and DCs incubated with CTX, CB, and LPS compared with that obtained in cultures of CD3^+^ T cells and DCs incubated only with LPS. Therefore, our results suggest a downmodulatory effect of CTX and CB on DC maturation.

The correlation between the modulatory effect of distinct pathogens or their products on DCs and consequent T cell activation or differentiation has been the focus of intense studies. In this context, Klaver et al. [52] showed that the soluble products of *Trichuris suis* are able to inhibit the maturation of DCs induced by LPS, as well as the production of proinflammatory mediators, such as TNF-*α*, IL-6, IL-12, and LT-*α*. It was also described that the soluble extract from *Schistosoma mansoni* eggs (SEA) inhibits the activation of immature DCs in response to TLR ligands, such as LPS or CpG.

In another studies, we also demonstrated that high molecular weight components (PI) of *Ascaris suum* mediate an inhibitory effect on the expression of costimulatory molecules and MHC-II on DCs and consequent ability to induce activation of OVA-specific T cells response. Furthermore, we observed that IL-10 is involved in this process [30]. Also, PI downmodulate the DC maturation induced by ligands of TLR2, TLR3, or TLR4. Furthermore, we verified that TLR2 and TLR4 are not involved in the modulatory effect of PI on DCs [53].

Chen et al. [54] also showed similar inhibitory effect of dextromethorphan (DXM) on LPS-induced DC maturation. In another work, it was demonstrated that trypomastigotes (Tp) of *Trypanosoma cruzi* exert downmodulation of costimulatory molecule expression as well as the activation of MAPK (ERK and p38) and NF-*κ*B pathways in LPS-stimulated DCs, in contrast with increased IL-10 production and an impaired ability to induce lymphoproliferation [55]. Similar results were published by Hamilton et al. [56] demonstrating that *Fasciola hepatica* tegumental antigen (FhTeg) suppressed the production of proinflammatory cytokines (IL-6, TNF-*α*, and IL-12), expression of costimulatory molecules and activation of NF-*κ*B and MAPK pathways (ERK) in these DCs incubated with LPS. In view of these observations and our results, we suggest that the CTX or CB effect on DCs is fully correlated with the downmodulatory effect on these molecules on adaptive immunity as previously described.

Considering the previous works showing the role of FPRs on the inhibitory effect of CTX on inflammatory reactions and also macrophage activities [29, 33, 37], we analyzed the involvement of these FPRs on the modulation exerted by CTX and CB on DCs. Therefore, we used Boc-2 that is an antagonist of FPR, FPR2/ALX, and FPR1, widely expressed by human and murine cells (monocytes, neutrophils and DCs) and is used to block the binding of different ligands such as lipoxins [38, 57–59].

We observed that the preincubation of DCs with Boc-2 abrogated the inhibitory effect of CTX and CB on the expression of costimulatory and MHC-II molecules as well as the secretion of proinflammatory cytokines induced by LPS. In agreement with our results, Kang et al. [60] demonstrated that the activation of FPR and FPRL2 by synthetic peptide Trp-Lys-Tyr-Met-Val-D-Met (W-peptide, an agonist of FPRs) inhibits LPS-induced human DC maturation. Furthermore, in murine model of noneosinophilic asthma model induced by intranasal sensitization with LPS plus OVA, it was verified that the activation of FPRs by this synthetic W-peptide results in inhibition of lung inflammation after OVA challenge. In addition, an impairment of the DC maturation and migration from the lungto-draining lymph nodes as well as IL-12 and IL-6 secretion was observed, which resulted in reduced Th1 and Th17 cell responses that are predominant in this asthma model.

Besides that the mechanism involved in the process of DC maturation is not completely clarified, our data using CTX and its CB subunit, immunomodulatory molecules, demonstrate consistently that FPRs play a role in the DC biological activities.

## 5. Conclusion

In summary, our results show for the first time the direct effect of CTX and its CB subunit on DCs, which represent a crucial link between innate and adaptive immunities. Thus, these results support the hypothesis that the effect of CTX and CB on DCs, via FPRs, contributes to its ability to inhibit the adaptive immune response.
